# Fractures of the Cervix Femoris

**Published:** 1823-12-01

**Authors:** 


1. *Practical Observations in Surgery.* By HENRY EARLE, F.R.S. Assistant Surgeon to St. Bartholomew's Hospital. Octavo, pp. 229, with plates. London, 1823.

2. *Observations on Fractures of the Neck of the Thigh-Bone, being an Appendix (the 2d.) to the Work on Dislocations and Fractures of the Joints.* By Sir ASTLEY COOPER, Bart. F.R.S. Surgeon to the King, &c. &c. Quarto, pp. 52, with plates. London, 1823.

EVERY man who comes before the public as an author, must expect criticism—nay detraction, if he excites attention at all—while he who stands forward as a commentator or critic on the works of others, plays a still more dangerous game; and is almost certain to repent of his enterprize if he permit any passion of the heart to interfere with the cool deductions of the head. This observation is the result of the general experience of mankind in all ages, and is not at all more applicable to the writings at the head of this article, than to the every day productions of the press. The authors, indeed, before us are conspicuous marks—one of them more especially—for the arrows of criticism—and the controversy between them, even were it on a much less important subject than the fracture of a bone, will be sure to furnish a prolific topic of conversation for the gossips of the profession. But this is not all. The controversies of eminent men, like the quarrels of kings, are not confined to the individuals immediately concerned. They become the centres of systems, around which other bodies, of various densities and magnitudes, revolve in formidable gyrations, or meet in hostile collisions. As far as the principal belligerents are concerned, it is impossible for any periodical writer to be neutral—because, the motto on both sides will doubtless be—"he who is not for me is against me." But there is a third party concerned—the public—the great body of the public, far removed from the personal influence of either of the other parties; and, to them, dispassionate reasoning and equitable statements are rather more agreeable than the most laboured or eloquent harangues. Whether, in the following exposition, we shall be able to divest ourselves entirely of prejudice or partiality, we really do not know, and, consequently, dare not affirm—but this we aver, that we will give no opinion that is not the offspring of conviction in our own minds. We may err in judgment, but we will not intentionally lead astray.

Our readers are aware that, in the 3d volume of this series, pages 628—640, we gave a very ample analysis of Sir Astley Cooper's Observations on Fracture of the Cervix Femoris, and that we returned to the subject at page 225 of the present volume, with some additional facts and observations. Since that period, Mr. Henry Earle has published a volume, the principal object of which is to controvert some of Sir Astley's positions—and this publication naturally gave origin to the second Appendix now on the table before us, from the pen of Sir Astley Cooper. We may observe, in limine, that an alloy of *feeling*, we had almost said *passion*, pervades some portions of both these productions, but especially Mr. Earle's, which badly harmonizes with a scientific discussion. It is unquestionable too, that the unhappy example was set by the junior controversialist, as is evident to the most superficial observer. Indeed, Mr. Earle must have been conscious of this himself, when it was rather late; otherwise he would not have penned the following apology in the preface.

"On an attentive and *dispassionate* revise of the following work, since the sheets have been printed off, some expressions appear to have escaped me in the warmth and hurry of composition, which may possibly admit of a construction very different from my wishes or intentions. I am anxious, therefore, most distinctly to disclaim the slightest feeling of disrespect towards the author whose work I have reviewed; and should any passages appear to the reader as too strongly expressed, I must entreat his indulgence in referring them to the ardour of a person writing on a subject which has occupied much of his attention, who was anxious to establish opinions which he has long entertained, and to introduce a practice which he hopes will be found beneficial."—*Preface, p.* x.

In all scientific discussions *passion* invariably weakens the cause in which it is enlisted—but when it is betrayed in the commentaries of one individual on another in the same line of profession, it excites emotions in the byestander by no means favourable to the aggressor. But, notwithstanding this aggression on the part of Mr. Earle, from whom, as a young man, it comes with a very bad grace, we by no means excuse Sir Astley for not only retorting in kind, but with an occasional tone of severity quite foreign to the general tenor of his character, and totally unnecessary in a man of his rank and influence in the profession.

The extensive series of extracts given in our former analyses will save us much repetition here; and, therefore, we shall only dwell on those points which are of most practical importance, and on which the main questions of the controversy hinge—begging our readers to keep our former analyses before them, for reference, where the original work is not in their possession.

Mr. Earle, at page 2 of his work, has given a sketch of the anatomy of the thigh-bone in the adult, and there stated that the depression for the insertion of the ligamentum teres is in the centre of the head of the thigh-bone. Sir Astley questions the correctness of his anatomy, and asserts that, for the very best physiological reasons, the depression is *not* in the centre of the head of the bone. Speaking of the round ligament, Mr. Earle says, page 7,—" This ligament is so disposed that the luxation of the femur on the obturator foramen is the only one which can happen without rupturing it." On this passage Sir Astley remarks—

" If it be meant that when the muscles and capsular ligament are removed, the head of the bone can be drawn over the edge of the acetabulum, towards the obturator foramen, every tyro in anatomy knows it; but if it be intended to state from this structure, that the dislocation in the obturator foramen, happens without rupture to this ligament, dissection proves that such opinion is erroneous."—*App.* 8.

At page 157 of Mr. Earle's work, we find the following passage:—

" If the capsular ligament was the principal support of the fractured portion, it is probable that retraction would be much more frequent, and to a greater extent, than it usually is; as, strictly speaking, the capsular ligament could afford but little resistance, both on account of its looseness and want of strength. What ligamentous band Sir Astley alludes to, I am rather at a loss to understand. There are, indeed, some few fibres of aponeurosis, which extend over the surface of the ulna at the base of the coronoid process and olecranon, but I have not been able to detect any distinct ligament on dissecting the joint, nor do I find any such mentioned by Soemerring, or any other author." 158.

To this Sir Astley replies:—

" If the author will have the kindness to call upon me, I will shew him as follows:—A band of ligamentous fibres crossing from the side of the coronoid process to the olecranon; and upon the radial side of the ulna, the upper portion of the coronary ligament of the radius passing from the side of the olecranon towards the neck of the radius. If the olecranon be broken off, and these ligamentous fibres be left entire, the olecranon will remain still united to the ulna by means of these ligamentous productions, which I should not have noticed but for their influence on fractures of this bone." 9.

A plate is introduced by Sir Astley Cooper illustrative of this band of ligamentous fibres. From anatomy we now come to pathological surgery. Speaking of the causes of fracture of the cervix femoris, Mr. Earle observes:—

" The neck of the thigh-bone is so protected by the surrounding soft parts and the trochanter major, that it is completely defended from the immediate action of external violence; and, consequently, from any direct fracture. It follows that it must be broken by a contre-coup, either by a fall on the trochanter, the feet, or knees. The former of these is by far the most frequent cause; although, on a superficial view, it may not appear the most probable. Desault says, that, in his practice, twenty-four cases in thirty were produced in this way. The experience of Sabatier and Richerand proves the same fact."—*Earle*, *p.* 20.

But, *audi alteram partem*.

"These observations," says Sir Astley Cooper, "are quite contrary to my experience, for fractures of the neck of the thigh-bone within the capsule, have been often produced by violence which acted perpendicularly; or they have been occasioned by a sudden rotatory motion of the pelvis upon the thigh-bone; the violence produces its effect by acting with the advantage of a lever upon the neck of the bone, but in fractures external to the ligament, they are produced generally by severe blows from falls upon the trochanter itself; in the former case, the patients are often sensible of the injury before they fall, and fall in consequence of it, and therefore, the accident is commonly imputed to the fall; but in fractures external to the ligament, the blow forces the trochanter towards the neck of the bone, and the trochanter splits and receives the cervix into its cancelli.

"With respect to Dessault, whose practice, for a short time, I had an opportunity of witnessing, and for whose character I have the highest respect, like some of our English surgeons, he confounded the three fractures of the upper part of the thigh-bone, under the general terms of "fracture of the neck of the thigh-bone."—*Appendix*, *p.* 10.

From attentive consideration we conceive, that the foregoing discrepancy of opinion is produced by Mr. Earle's applying the same causation to the fractures at different parts of the cervix, as described by himself in the 19th page. He acknowledges, at page 21, that "a large majority of the cases which he has witnessed, had been caused by falls on the pavement, &c."—and, occasionally, by falls when the person was carrying a heavy burthen. In these cases, and especially in people not advanced in years, the fracture would usually be external to the ligament, as Sir Astley Cooper has properly observed. We now come to the subject of Diagnosis.

"Where a person," says Mr. Earle, "previously in full possession of the locomotive powers of his limb, after the receipt of an injury, and particularly after a fall upon his trochanter, becomes suddenly deprived of that power; accompanied with a remarkable consciousness of incapacity in the injured member; and when, from the position and direction of the limb, it is obvious that there is no dislocation, a strong presumption must arise that a fracture has taken place. In such a case a surgeon is *fully warranted to act on such a*

*suspicion*, and to treat the case as a fracture, without subjecting his patient to painful examinations, to gratify his own curiosity: the consequences of which, in addition to the pain inflicted, may be most injurious, and often render complicated, what, in the first instance, was simple.

"I cannot quit this subject without deprecating, in the strongest terms, the cruelty and impropriety of what is termed 'satisfying yourself that there is a fracture, and its precise situation.'" 23.

And at page 24—

"To return, then;—This symptom of a total and sudden privation of the power of motion, after a fall on the trochanter, should always be regarded as diagnostic of a fracture."—*Earle, p.* 24.

To the above passages we would urge objections of a prudential, chirurgical, and ethical nature. In the first place, we conceive that, even Mr. Earle himself will hardly deny that this sudden privation of locomotive power, after a fall upon the trochanter, may sometimes take place without either fracture or dislocation. And in what situation will that surgeon's character stand, who pronounces the serious verdict of "fracture of the neck of the thigh-bone" on a man who, in two or three days afterwards shall be walking about his business?—This is no imaginary case. We have repeatedly seen men fall from heights and upon hard bodies, and remain for many hours incapable of making any use of the limb, yet without there being any serious injury of the joint. It is true, we were not so very delicate as to take it for granted that there was a fracture of the neck of the femur, without examination. We were obliged to give a report of the nature of each case —and we first satisfied our "*curiosity*"—a laudable one, we hope—respecting the true state of affairs.—But again: we see in more than one place, in the above extract, that "*the cruelty and impropriety*" of examining a limb, so as to ascertain the existence or non-existence of fracture, is emphatically denounced. Now Mr. Earle either means to impute this practice to Sir Astley Cooper, or he does not. If he does not, why bring it forward in a formal critique on Sir Astley's practice? But that Mr. Earle *did* mean the charge of "cruelty and impropriety" to directly apply to Sir Astley, is, to our apprehension, most evident, from the following passage in page 84.

"Now, if the generality of Sir Astley's patients, and particularly those in Guy's Hospital, have had all these indications of fracture demonstrated upon their persons, it is not difficult to understand the great degree of shortening of the limb, and destruction of the fibrous periosteum, which he has found in fractures within the capsule. I have already commented on the worse than inutility of such examinations in another part of this work, and only revert to the subject to explain one source of failure in Sir Astley's practice." 85.

Such a serious and sweeping charge against the practice of a surgeon, without any proof being adduced, is, in our minds, a breach of all medical ethics, and highly censurable in any member of a liberal profession. Of this charge of rupturing ligaments, &c. during an examination, we say Mr. Earle has adduced no proof. On the contrary, he has detailed cases, and quoted authorities, which very clearly prove, what, indeed, every anatomist well knows, that the living tissues, and especially the ligamentous structures, are not of such gossamer fabric as to be easily torn by even pretty rude examinations of the surgeon. At page 30, the case of Daniel Spilling is related, whose cervix femoris was fractured, and who was not only "examined with *great freedom, and for a considerable length of time*," in various postures, but was subsequently directed to lie on his right side with the thigh bent on the pelvis—a "position that gave him great pain in the hip, and soon became intolerable." Our author also remarks, that very little attention was paid to the limb, (in Bartholomew's Hospital) and the man died of constitutional disease, on the 12th day after admission. Now here was a case, of course, to exemplify the rough examinations said to be used by Sir Astley Cooper. Yet, on examination after death—"the fractured surfaces accurately fitted to each other, and were retained in their proper relative situation by a portion of the reflected layer of the fibrous and synovial membranes, of about an inch in extent, which had fortunately *escaped being torn, notwithstanding all the examinations which were instituted after the accident, and the total neglect of any apparatus to restrain the motions of the limb*." 32. "I have met, (says Mr. Earle) with two other cases, in which considerable portions of these membranes remained undivided, notwithstanding the *great force* which was employed in ascertaining the nature of the accident." Our author quotes Delpech, who asserts, that the reflected synovial and fibrous expansion is rarely torn, at least to any considerable extent.—"Cette expansion est rarement rompue, du moins en grande partie." These cases and these quotations prove at least that, in those few fortunate instances where the reflected membrane is *not* torn by the accident which produced the fracture, it is not likely to be torn afterwards by the mere examination of the limb. Not that we attach any importance to the assertion of Delpech that the reflected capsule is rarely torn—because it is well known, that Desault and other French surgeons have generally confounded the three different kinds of fracture of the cervix and upper part of the thigh-bone, denominating them all "fractures of the neck of the bone." We maintain, therefore, that it is contrary to the best principles of medical ethics to attribute the rupture of ligaments and membranes, and the non-union of bones to the improper manual examination of one of the ablest surgeons of the age we live in, merely because the general experience of the profession does not tally with a favourite proposal or doctrine of Mr. Earle. We certainly do not much wonder that this insinuation should have called forth some expressions of unusual warmth from Sir Astley Cooper at page 11 of the Appendix; though we are confident that he would have best consulted his own dignity, if he had merely quoted the passage, and appealed to his brethren as to its moral tendency—or if he had confined himself to the following sentence,

> "With respect to my own cruelty or inhumanity, *particularly to the patients in Guy's Hospital*, God forbid that I should be the advocate of a cruel, coarse, or unnecessary examination, and no one has a right to accuse me of it; all I have recommended may and ought to be done with gentleness; ask my greatest enemy, and let his answer decide, whether the poor of London, and the patients in Guy's Hospital, are or are not anxious to place themselves under my care." 14.

We can aver that the distinguished surgeon in question is not less revered by his pupils than beloved by his patients in Guy's Hospital, if any credit can be placed in the physiognomies of both classes. But this by the way.

The next point of discussion is the retraction of the limb. Mr. Earle asserts that, in every instance he has examined, the retraction did not exceed from half an inch to one inch, except where the neck was entirely absorbed.

> "I have stated it," says Sir Astley Cooper, "to be from one to two inches shorter than the other limb; and, from having since seen a case in which it was more than two inches, I extended it to two inches and a half. But, instead of this statement being greater than it ought, I have now to add, that in Mr. Langstaff's collection there is a preparation of the fractured neck of the thigh-bone within the capsule, taken from a man named Campbell, aged 82, in whom, Mr. Langstaff assured me the shortening was four inches; and, to convince me of this, he sent for the shoe which the man had worn after his accident, and which I measured: the heel of the shoe was two inches and a half in thickness, and he wore some pads in the shoe, which raised his heel one inch and a half higher to bring it even with the other leg; therefore if I were to alter the sentence, it would not be in the way our author wishes, but by making the utmost difference in length four inches. The usual state is, that the heel of the injured limb rests in the hollow between the malleolus internus and tendo achillis of the other leg. I know that there are examples in which the limb has been shortened only half an inch, but these are exceptions to a general rule, and they depend upon the mode in which the fracture has happened; for a fork is sometimes formed in the trochanter minor, which receives the neck of the bone, and prevents its farther descent." 16.

Sir Astley further observes that, in fracture of the neck of the thigh bone *external to the capsule*—when the trochanter splits, and the neck of the thigh-bone is received into its cancelli, the degree of shortening of the limb is very slight, being generally from half to three quarters of an inch. The reason is obvious—for the head of the bone passes outwards more than it descends. Our author has lately inspected a case of this kind with Mr. Key. The moment he saw it he pronounced it to be a fracture external to the capsule, from the limb being so little shortened—not three quarters of an inch, and other circumstances. The neck of the bone was found forced into the cancelli of the trochanter major. The parts are preserved for inspection, and Mr. Key vouches for the degree of shortening.*

Mr. Earle next adverts to the case in Mr. Langstaff's Museum, in which there are two fractures, one within, the other external to the capsule. The former is united (though slightly) by ligament, the latter by bone. The following passage, from Sir Astley's appendix, will give the reader a sufficient idea of this part of the controversy.

"From reading my account of a preparation, contained in Mr. Langstaff's excellent Museum, of fracture of the neck of the thigh-bone within, and another external to the capsular ligament, it is said in the work before me, page 40, 'I was led to make a very particular examination of this preparation, and the inferences which I draw from such examination are very different to those at which Sir Astley has arrived.'

"The only conclusion I drew was, that the one unites by bone, and the other by ligament.

"He says—'In the first place, I observed that the fractured surfaces within the articulation were very accurately opposed to each other, and, consequently, that very little shortening could have taken place.

"To this I answer, that not one word was said respecting their being opposed to each other or not, and therefore no conclusion was drawn by me about it; and as to ascertaining the shortening of a

---

* As for the cloud of authorities from the other side of the Channel which have been cited by Mr. Earle, we confess that they appear to darken rather than illumine the subject—since, as we before stated, (see Mr. Cross's sketches,) the French surgeons have made little or no distinction between fracture within and external to the capsule.

limb, preserved in a glass, and separated from the surrounding bones and muscles, no correct opinion could thus be formed upon the subject.

" 'Secondly: (says Mr. E.) I perceive that very firm ligamento 'cartilaginous union had takeh place.'

" I would ask, is this the *ossific union* for which the author contends? The truth is, that I have seen a number of cases of fracture of the thigh-bone within the capsule, but, as will be seen in the plate, never less union, even by ligament, than in this preparation. Mr. Langstaff is a most liberal man, and will be happy to shew the preparation to any person anxious to see it.

" 'Thirdly:—It was evident, that a considerable portion of the 'reflected membrane was not torn through.'

" I have drawn no conclusion from the preparation in that respect; but I could wish Mr. Langstaff to be asked if any portion of the reflected membrane remains.

" 'Fourthly:—It appeared very clear, that the fracture within 'the articulation would prevent the motion of the pelvis from being 'communicated to the fracture external to the capsule.'

" I do not see this; the motion of the pelvis must be communicated to the insulated portion of bone through the medium of the capsule.

" Here then the conclusions are entirely the author's, and not mine; for I drew no other than that the internal fracture did not unite, and that the external united; and with respect to the accuracy of description, I wish the reader to consult the preparation, as well as plate 11, which is a faithful representation of it."* 20.

The period of life at which these fractures happen has been a prolific source of animadversion on the part of Mr. Earle—and that evidently from some obscurity in Sir Astley's statements, by which the cases he designed for exceptions have been misconstrued for the general rule. We therefore acquit Mr. Earle of any thing like intentional misrepresentation on this point—while the original rule laid down by Sir. Astley stands, according to our minds, precisely where it did, and equally as strong as ever.

" I have now been," says Sir Astley, " thirty-nine years at St. Thomas's and Guy's Hospitals; and, for thirty years, have had more than my share, and much more than I merited, of the practice of London. We have eight hundred and fifty patients in our Hospitals; and I believe that, in the two hospitals, eight cases of fractures of the upper part of the thigh-bone occur in each year; but, in order to prevent my exceeding the average number, I will consider them only as five per annum; thirty-nine, multiplied by five, produce one

---

* Mr. Earle has since acknowledged that he entirely mistook the preparation in Mr. Langstaff's museum, describing one preparation for another.

hundred and ninety-five; add to these one case only in each year, in my private practice of thirty years, they will collectively amount to two hundred and twenty-five cases; now, in that time, I have only known *two cases* of fracture of the neck of the thigh-bone within the capsular ligament *occur under fifty years of age;* one was in a patient aged thirty-eight, who had an aneurism of the iliac artery; and the other has been kindly shown to me by that excellent anatomist, Mr. Herbert Mayo. Our author states that he has 'examined, 'post mortem, three fractures of the neck of the thigh-bone within 'the capsular ligament in young people.' I shall request permission to examine these rare and curious cases, which our author has no doubt preserved." 21.

But having acquitted Mr. Earle of any intentional misconstruction of the three cases designed as exceptions by Sir Astley, we know not how to do the same respecting the passages marked in italics in the following extract:—

"And this is all the evidence (the three exceptions mistaken for general rules) adduced to prove that fractures external to the capsule occur in early life—that they are the result of great violence—*that they are not shortened*—and that *they are capable of ossific union.*" *Earle*, 50-1.

We shall give the answer to this passage in Sir Astley's own words.

"To the first, I have to state, that I have mentioned that fractures external to the ligament *occur under fifty years of age;* a great number of these cases which have fallen under my notice, have occurred under that period, and, therefore, a surgeon called to the bed-side of a patient who has an injury to the upper part of the thigh-bone, if he finds the age of the patient to be under fifty years, will, with a very rare exception, be correct in his judgment, if he concludes it to be either a fracture just external to the ligament, or one through the trochanter major. But I also mention that *both fractures occur in age*, and therefore no conclusion can be drawn between the two, in advanced age, but by the most careful examination. The three cases which I have given clearly shew this. If I had said these fractures external to the capsule only occur under fifty years of age, our author would have had some foundation for his remarks.

"I have stated that fractures directly external to the capsule, arise from greater violence than fracture within the capsule; for my experience has taught me, that a very slight accident generally occasions the fracture within the capsule, and a violent blow, or fall, the other: the first is an accident upon which the fall often succeeds, the other is generally the consequence of that fall; many of those within the capsule which I have witnessed, were produced by the person's slipping from the curb-stone to the road-way,*—not that I mean to

* "Slipping from the curb-stone to the road-way produces a violence in a perpendicular direction; falling against the edge of the curb-stone produces often the fracture external to the capsule."

deny, that a fall will, and does occasionally, produce a fracture within the capsule, or that in a very old person, a fracture may occasionally happen in any part of a bone, from, comparatively, a slight cause to that which produces it in the young.

"It is also said, that 'I have observed they are *not* shortened;' whereas, I have expressly said, '*The limb is shortened,*' but not to the same extent as in the internal fracture, for the difference in the length of the limbs rarely amounts to an inch; its extent depending upon the obliquity of the fracture, and upon the degree of laceration of the surrounding parts admitting of a greater or less retraction of the muscles.'

"It is further said, 'That I give no other evidence of the fractures 'external to the capsule, and those through the trochanter, being 'capable of ossific union.' How could such a statement be made, when I have given three plates of the ossific union of the cervix external to the capsule, and one of the trochanter united. (See the plates in the first and second edition of my work on Dislocations and Fractures.") 24.

Mr. Earle next enters into a critical examination of the reasons which Sir Astley Cooper has adduced against the probability of ossific union within the capsule. First, however, he places poor John Bell in a situation of ridicule, for having given contradictory evidence on this point. Now, as Mr. Bell's statements are confessedly contradictory, and consequently unavailable by either party, why does Mr. Earle "draw forth his failings from their dread abode," and cast a stigma on the memory of the illustrious dead—now no longer rble to reply—apparently for no other purpose than the gratification of a censorious appetite? Surely this is bad taste. But to proceed.

Sir Astley had stated that if the broken extremities of a bone in any part of the body be kept much asunder, ossific union is prevented. While Mr. Earle admits the truth of this, as far as the neck of the femur is concerned, he doubts the correctness of the statement as a general position, referring to Mr. Dunn's case, commented on by us at page 633 of the 3d volume of this series. We have seen some preparations lately which appear to shew that where a portion of one bone is cut away, say the tibia, the adjacent bone or fibula will sometimes throw out an osseous projection between the separated extremities of its neighbour, and thus produce a union, or continuation of bone. But this will not apply to the neck of the femur, which has no such friendly ally to lend it ossific assistance.

Sir Astley promises, in the 3d edition of his work on fractures, five different examples of non-union of bone, from separation. He is well aware, indeed, that two or three inches of the femur or os humeri may be removed, with ultimate union of bone—*if the broken ends are suffered to be drawn together by the action of the surrounding muscles.* In illustration of this, an interesting case is published by Sir Astley, as communicated by the present Mr. Benjamin Bell of Edinburgh, a short abstract of which we shall present to our readers.

A miner, 35 years of age, while exploding a rock, was struck by a fragment of stone which shattered the tibia and fibula of the left leg about four inches below the knee. Four large loose pieces of bone were extracted immediately after the accident. These portions, when united, formed about six inches of the entire cylinder of the tibia. The sides of the wound were drawn together and retained by sticking plaster, and the limb kept in a secure but easy position. The wound healed by the first intention, but a subsequent abscess formed, from which was discharged a portion of bone, thought to be a fragment of the fibula. When examined, thirteen months after the accident, the leg appeared to be about two inches shorter than the other. A large cicatrix occupied the fore and middle part of the shin—the patient could extend the leg and stamp on the floor with considerable force; "*but the leg was to a certain extent flexible, and could be slightly bent by the hands in four different directions.*" "It seemed to me, (says Mr. Bell) as if the space between the two ends of the fractured bones had been filled up with a sort of ligamento-cartilaginous matter, resembling that found in cases of fracture of the neck of the femur external to the ligament," &c.

In respect to Mr. Dunn's case, of which Sir Astley has received a cast from Mr. D. himself, some experiments are to be detailed in the 3d edition, explanatory of that very curious phenomenon.

Sir Astley Cooper had stated that an increased quantity of synovial fluid is secreted in cases of fracture of the cervix, from the augmented determination of blood to the capsular ligament and synovial membrane—and that this superabundance of synovia distends the capsule, and thus prevents the contact of the bones, by pushing the upper part of the body of the thigh-bone from the acetabulum. Mr. Earle observes upon this that, as death rarely takes place soon after the accident, and as it is allowed by Sir Astley that the superabundant synovia is, after a time, absorbed, so "it is more than probable that this fact requires the confirmation of actual observation." Sir Astley has referred Mr. Earle to a very unexceptionable authority on this point—namely, to himself (Mr. E.)—who, as our readers may see, in p. 32 of Mr. E.'s book, punctured the capsular ligament on the 12th day, and let out "*an ounce of bloody synovia.*" It is true, the bones were *not* separated here, *because* a portion of the reflected membrane was left untorn. But the existence of the superabundant fluid being conceded, Mr. Earle's ingenuity makes its presence contribute to the approximation rather than separation of the bones, in the following manner.

"To illustrate this, and place it in the clearest point of view, *we will suppose* an oblong bag rather loosely applied over a cylindrical portion of wood, and firmly fastened at the distal ends of the same; *then* let the wood be divided transversely near its centre, and the loose state of the bag will in that case admit of a certain degree of separation between the two portions: but distend the bag with fluid, taking care to keep the divided ends of the wood in their proper relative situation, and it will be proved to demonstration that the two pieces of wood will be brought into contact with each other; and the greater the degree of distention, the closer will be the contact to which they will be brought; because the process of distention will have the effect of expanding the sides of the bag, which alone can yield to the pressure of the fluid, and consequently of approximating its two ends; which being fastened to the distal ends of the two pieces of wood, must force their divided surfaces into closer contact." 75.

We are rather at a loss to conceive how Mr. Earle divides the piece of wood *after* the ends of the bag are firmly fastened over it. The whole experiment, however, is acknowledged to be a *supposed* one—and from this supposition let him turn to the knee under an extension of its ligaments from an increased secretion of synovia, when he will see its bones unequivocally separated.

Mr. Earle reverts again and again to the hypothesis, for such we must regard it, that Sir Astley's examination of the limb tears the reflected membrane, and thus prevents the chance of bony union. We shall not again enter into controversy on this point, having proved by the very cases adduced by himself, that the roughest handling and entire neglect of treatment afterwards, did *not* lacerate the membrane, or separate the ends of the bones.

Mr. Earle next objects to the conclusions which Sir Astley Cooper came to, from his experiments on animals—and that upon three grounds—the opening of the capsular ligament rendering the fracture a compound instead of a simple one—the laceration of the reflected membrane—and the unrestrained motion of the limb afterwards. The following extract will convey Sir Astley's replies to these objections.

"The next observations relate to my experiments on animals, which prove that the head of the bone, even in healthy animals, will not unite with the cervix; and which are objected to upon the ground that they were compound fractures. Now these fractures were not compound in the true sense of the word, for they were united by adhesion and not by granulation; the wound which was made through the integuments was valvular, and healed immediately. A compound fracture unites by granulations, and a simple fracture by adhesion; but here the external wound almost immediately adhered, and, consequently, it did not heal by granulation. They are also objected to on the ground of the ligament being cut through. In one of these experiments the capsular ligament was opened: in the other it will be seen it was not: both were broken by a blunt instrument. The two preparations which I have preserved were the only examples in which the experiment was complete, as regards the transverse fracture; but I have two other interesting preparations derived from these experiments. I also made a great number of others, in which the fractures continued compound; and in each of these, the head of the bone either became absorbed, or was discharged by ulceration, and I never could succeed in uniting the head to the neck of the bone. I have since divided the neck of the thigh-bone in a dog, and the head of the bone was three-fourths absorbed. By way of contrast, I have since divided the bone externally to the capsule, in five instances, and have preserved the bones; the wound united by adhesion, and every bone has been healed by ossific union; the natural inference is, that fractures within the capsule do not unite by bone, but the fractures external to it readily do so. As to the bearing upon the limb, or its weight having any influence to prevent union in these animals, I have only to observe, that the muscles become contracted, the limb drawn up, and the animal cannot bear upon it for several weeks.

"My friend, Mr. Brodie, has furnished me with the following account of an experiment he made, and which fully confirms my observations.

"Dear Sir,

"The circumstances of the experiment, which I mentioned, were briefly these:—The tibia of a guinea-pig was broken at the lower end. A month afterwards the animal was killed. On dissection I found a fracture extending across the tibia, transversely, and so close to the ancle-joint, and it was situated at that part of the bone which is covered by the reflected layer of the synovial membrane. The synovial membrane itself, and the ligaments of the joint, appeared to have been very little injured, and the broken surfaces had remained in good apposition; nevertheless, there was not the smallest union of them, either by bone or ligament, and there had been no formation of callus round the fracture. The bone in the neighbourhood of the fracture, had become compact and hard, in consequence of the ossification of the medullary membrane lining the cancelli.

"I am, Dear Sir,

"*Saville Row,* "Your's truly,

"*August* 16*th*, 1823. "B. C. Brodie." 31.

Mr. Earle has given a short abstract of Mr. Colles' eleven cases of fracture of the cervix, and makes objections to most, if not all of them, as not bearing properly on the subject in litigation. One of the prominent objections rests on the circumstance of several of them being dissecting-room subjects where—"*as nothing is known of the treatment, no conclusion can be drawn from the case.*" Surely Mr. Earle will not object to the same rule being applied to himself—unless, as sometimes happens, he has one system of evidence for himself and another for his adversary.

By this time the reader will be ready to agree with Mr. Earle that "he has hitherto only adduced *arguments* in opposition to the reasons which have been assigned for the want of bony union, and that the *onus probandi* rests with him to shew that bony union has taken place." It certainly does—and we now proceed to inquire how he has exonerated himself.

"That such an occurrence, without any lameness, or perceptible shortening of the limb, has happened several times in the course of my experience, I am perfectly satisfied of in my own mind; but as the individuals are either now living, or have died without affording me an opportunity of ascertaining the actual state of their limbs, I cannot reasonably expect to gain implicit credence to all the testimony which I might offer upon this subject." 95.

As this is a species of evidence which Mr. Earle would be the last person in the world to admit, we must beg leave to consider it "as dust in the balance," and to pass it over.

We now come to evidence from dissection. One of the nurses belonging to the hospital, aged 74, was knocked down in the street by a horse in the year 1808, by which accident she had the cervix femoris fractured. During the first three weeks little or no attention was paid to the patient. Mr. Earle then placed the limb in a proper situation (having even then distinctly felt a crepitus,) and put the patient into the double bed, in which she was kept seven weeks. She died at the end of thirteen weeks from the accident, and on dissection there was found a transverse fracture of the cervix, within the articulation, apparently united by bone. When boiled, however, "*the union became loosened.*" We shall allow Mr. Earle all the advantage which this case can afford his cause, without a single comment.

It is on the following case that Mr. Earle appears to rest the main body of his evidence.

"A very interesting case of bony union and osseous deposit within the articulation is recorded by Christopher Henry Erndleus.* The following is the description which he gives of the nature of the accident:—'Collum vel cervicem ossis femoris a violentiâ externâ frangi frequentius accidit, quam a plerisque creditur; ossea autem vel vitrea (uti à quibusdam appellatur) lamella in hâc parte tenuis-

* "Relatio Itineris Anglic. et Batav. p. 86."

sima est, et quamvis communis sit opinio, ubi patientes de dolore et motu abolito in his partibus conqueruntur, *exarticulationem vel luxationem ossis ischii accidisse, falsa tamen, imo propter incredibilem ligamentorum ac tendinum circumjacentium crassitiem ac robur, impossibile exarticulationem tali modo luxari.* Talem fracturam his meis oculis vidi et manibus palpavi in cadavere fœminæ nosocomei muliebris Amstelodamensis sociæ in quâ tractu temporis, fractura illa cervicis ossis femoris dextri per callum coaluerit iterum, *fœmina tamen exinde per omnem ætatem* ad mortem usque *clauda. Callus pollicis latitudine sub ipso capite magno ossis femoris extabat, nulla autem in ligamentis ac tendinibus musculorum læsio vel præter naturalis constitutio erat.*' " *Earle*, 98.

Mr. Earle observes that "*this case is the more valuable, as a simple narrative of the fact, without any controversial speculation on the possibility or not of union within the joint.*" 99. But we confess that the surgeon who seriously sets about proving that *dislocation of the head of the femur is a thing impossible*, would not be the umpire which we should choose in determining the nature of injuries of that part. Nor do we think the language of Mynheer Erndleus so unequivocal as Mr. Earle believes it. Does he mean to say that the callus stood out (extabat) an inch from the surface of the cervix, and that without producing any lesion or displacement of the tendons or ligaments in its vicinity? The case appears to us to be a *disease* of the bone, such as may be seen in any anatomical collection, and consequently we consider Erndleus to be totally deceived. But if Mr. Earle still stands out for the testimony of this surgeon, we have no objection—for he proves a great deal too much for him who quotes him. He unequivocally proves that by this bony union of the cervix (if it was such) the woman was lamed for life—"*fœmina tamen exinde per omnem ætatem ad mortem usque clauda.*' We think this is about one of the most unfortunate cases which Mr. Earle could have quoted in the whole corpus historicum of surgery.

Mr. Earle has found in Mr. Heaviside's collection " a bone evidently bearing the marks of having been *obliquely fractured just at the insertion of the capsular ligament*, in which there is a large osseous deposit round the base of the head." The position of the fracture (if it was one, which we very much doubt) deprives it of any influential bearing on the question under discussion.

Mr. Earle " reserves to the last," the " highly interesting specimen in the possession of Mr. Abernethy, found by Mr. Stanley in the body of a subject in the dissecting room." The subjects found in the dissecting room by Mr. Colles in Dublin, are, of course, to be counted as nothing—their histories being unknown. But the case is quite altered on this side of the Channel, and on Mr. Earle's side of the question. In this last instance it must "be admitted by the most sceptical"—although—"nothing is known respecting the case."

"As it is Mr. Stanley's intention to publish a description of these bones, I will only so far anticipate that gentleman's account by stating, that they were both found in the same subject; that the fracture on the right side was entirely within the articulation, and on the left side partially; that there was very little shortening of the limbs, arising only from the loss of obliquity in the neck; and, lastly, that the most perfect osseous union has taken place, which can be traced through the whole substance of the neck, in the different sections which Mr. Stanley has made." 100.

Having seen so many bones of old people where disease, and the efforts of Nature to repair the decay of our fabric, produce the similitudes of united fractures, although there never was a fracture, we beg leave to demur to the present proof, even at the risk of being denominated sceptical. Let us hear what Sir Astley Cooper has to say on this subject.

"The third proof of the union of the fracture of the neck of the thigh-bone is from the preparation of Mr. Stanley, upon the subject of which some observations are required. For Mr. Stanley I have great respect; I know him to be well founded in anatomy, and excellently qualified to make a good surgeon.

"The description which our author has given of this case is defective in two very essential points. In the first place, he does not say that in one of them the appearance of fracture was external to the capsular ligament, and, therefore, not applicable to his object. Secondly, he does not mention any thing of disease which existed in other joints.

"I do not mean to deny the possibility of the necks of both thigh bones in this subject having been fractured, because that point can only be determined by the history of the accident, and by a very careful and accurate examination of several sections of the bones; but I can shew that similar effects are produced by disease.

"The neck of the thigh-bone, in adult persons of middle age, has a close cancellated structure, with considerable thickness of the shell which covers it; but in old subjects, the cancellated structure of the shaft of the bone, which is formed of a coarse net work, loaded with

* "The author says, 'that the fracture on the right side was entirely within the articulation, and on the left side partially.' My memory may deceive me, but if I rightly recollect, the appearance begins between the trochanter major and capsular ligament above, and it extends to the insertion of the capsular ligament below. If this were fracture, and not a change from disease, upon my own principles, union would be readily effected, beginning, as it would, externally to the capsule."

adipose matter, is often extended into the neck of the bone, and the shell which covers it becomes so thin, that when a section is made through the middle of the head and cervix, it is found diaphanous; of this I have several specimens. As the shell becomes thin, ossific matter is deposited on the upper side of the cervix, opposite the edge of the acetabulum, and often a similar portion at its lower part, and thus the strength of the bone is in some degree preserved: this state may be frequently seen in very old persons. Mr. Steel, of Berkhampstead, one of the most intelligent surgeons, and most respectable men I know, gave me the thigh-bone of a person thus altered, whose age was ninety-three. When the absorption of the neck proceeds faster than the deposit on its surface, the bone breaks from the slightest causes, and this deposit wears so much the appearance of an united fracture, that it might easily be mistaken for it.

"Before the bone thus alters we sometimes meet with a remarkable buttress shooting up from the shaft of the bone into its head, giving it additional support to that which it receives from the deposit of bone upon its external surface.

"But another change is also produced from disease, of which the following is the history, and which directly applies to the subject before us:—

"Old bed-ridden and fat persons (generally females) are often brought into our dissecting room, with some of their bones broken (and more frequently the thigh-bone than any other) in being removed from the grave. If the cervix femoris of such persons be examined, it will be found that the head of the bone is sunken down upon its shaft, and that the neck of the thigh-bone is shortened, so that its head is in contact with the shaft of the bone opposite to the trochanter minor; and at the part at which the ligament is inserted into the neck of the bone, the phosphate of lime is absorbed, and a ligamento cartilaginous substance occupies its place; either extending through the whole neck of the bone, or partially, so that one section exhibits signs of it, and in another it is wanting. The bone, in some cases, is so soft and fragile both in its trochanter and head, that it will scarcely bear the slightest handling: the motion of the thigh-bones in the acetabulum is almost entirely lost, so that the persons could have little use in the lower extremities.

"During the last winter we had two instances of this alteration in the neck of the bone, and it is an appearance I have several times seen.

"In examining the body of an old subject, very much loaded with fat, in the dissecting-room of St. Thomas's Hospital, I found that the gentleman who had dissected one limb had cut through the capsular ligament of the hip-joint, and tried to remove the head of the thigh-bone from the acetabulum, but the neck of the bone broke under the employment of a very slight force; and, upon a further trial to remove it, the bone crumbled under his fingers. As the other limb was not yet dissected, I requested Mr. South, one of our demonstrators, to remove, with care, the upper part of the other thigh-bone; but although he used great caution in doing it, he could not remove the bone without fracturing the upper part of its shaft; but he succeeded in removing the upper part of the bone, so that it could be preserved; and of this bone I have given plates.

"We have here then a case in which the neck of the bone was absorbed so that the head was brought in contact with the trochanter, in which, most decidedly, there had not been a fracture, and in which the disease occurred in each hip-joint.

"Another case of the same kind was examined by Mr. South, during the last winter, which, so far as relates to the softened state of the upper part of the thigh-bone, was similar to the former; the head was spongy, the necks were shortened so that there was scarcely any remaining, the trochanter light in weight, spongy, and very large; and there was little if any motion in the hip-joint, and both limbs looked, at first sight, as if dislocated upon the pubes.

"But the best specimen of this state of the bone is the following, which I preserve with the most assiduous care, and value in the highest possible degree:—I have had for twenty years in the collection of St. Thomas's Hospital, the thigh-bone of an old person, in which the head of the bone had sunken towards its shaft. I have been in the habit of shewing this bone twice a year as a specimen how bones sometimes become soft from age and disease, and from the absorption of their phosphate of lime; and I had frequently cut, with a penknife, both its head and its condyles to shew this softened state. On sawing through its cervix, the cartilage, deprived of its phosphate of lime, had dried away in several parts, and the appearance was such, that a person ignorant of the change would have declared it to be a fracture: only, that in some sections the cartilage had taken different directions, and in some the bone was not yet entirely absorbed. We have also in the Museum of St. Thomas's Hospital, a skeleton in which both the thigh bones, and each os humeri, are, in a subject extremely altered by rickets, divided by similar portions of cartilage, in which no ossific matter exists.

"The plates which are appended will afford better ideas of these morbid changes than words can convey; and I hope Mr. Stanley will also give plates of his preparations; only *both* must be engraved, as without both the public cannot form a correct opinion." 42.

The last subject which is to be touched on is the treatment, and this need not occupy us long. We have not the smallest objection to Mr. Earle's bed, or to any apparatus which he may think conducive to the patient's ease, or the chance of bony union in the neck of the femur. But viewing as hopeless—and perhaps useless, or even injurious, the said bony union, we can only say, that were the accident to happen to ourselves, we certainly should not submit to one additional hour's confinement, with the view of attaining the object so much desired by Mr. Earle.

It has been remarked by one of our very respected cotemporaries, that Sir Astley Cooper has evaded the subject of treatment; but this is an oversight, as the following extract will amply prove.

"My belief is, that it is wisely ordained by nature, that this, and the other bones I have mentioned—the patella, olecranon, and extremities of the bones within the articulations, should not, as a general rule, unite by bone; for I find that if the neck of the thigh-bone be shortened, and falls towards the shaft of the bone, the person is unable to move his hip-joint, and becomes, what is vulgarly called, bed-ridden; the cause of this is easily explained: for when the cervix is shortened, the trochanter falls upon the acetabulum, and the play of the thigh-bones become destroyed. In an ossific union of the cervix femoris, if such should ever occur, as the neck of the thigh-bone is greatly shortened very soon after the accident, the same effect would be produced, and the play of the limb be destroyed; more especially when the callus, by which the union would be produced, must occupy a considerable part of the acetabulum, and the patient's state would probably be much worse than in that of ligamentous union.

"It has been said, that I have a strong prejudice upon the subject of union of the fractured neck of the thigh-bone, but this I cannot allow. When I began practice, I made use of all 'appliances and means to boot' to produce an ossific union, but finding I could not succeed in a single instance, and that my patients suffered not only in health, but from sloughing sores upon the back, which sometimes destroyed them, I gave up the attempt, and now aim only at tranquillizing the parts to prevent inflammation, and thus lessen the danger of the injury; and I endeavour, as soon as possible, to restore to the patient an useful limb by crutches,—by a high heeled shoe,—and by the aid of a stick,—until he can walk without one.

"I have witnessed the practice of Mr. Cline, Mr. Chandler, and Mr. Birch; of Mr. Warner, Mr. Lucas, Senr. Mr. Forster, and Mr. Lucas, Junr. in our two hospitals of St. Thomas's and Guy's, and I have seen the practice of a great number of surgeons in London, yet I never saw one of these gentlemen succeed in uniting a fracture of the cervix femoris within the capsule, although all variety of means were tried which their minds could suggest. Were these surgeons all prejudiced?

"The treatment which ought to be pursued in fractures of the upper part of the thigh-bone is as follows:—

"In fractures within the capsule, the limb is to be placed over a pillow, bent under the knee, from a fortnight to three weeks, until the inflammation in the joint, and the irritative fever have subsided; and when the patients are able to rise, the sooner they use crutches the better, because the more quickly do they recover. They are to bear gently on the foot at first, and gradually more and more, until the ligament becomes thickened and the muscles increased in their powers, so as to enable them to walk with a stick: a high heeled shoe is next advised them, by which the halt is much diminished; and then, if the person be not of great weight, the stick may be discontinued.

" In fractures of the neck of the bone external to the ligament, when the cervix is thrust into the trochanter, the mode of treatment consists in placing the patient over the double inclined plane* and a band or girt is to be buckled around the pelvis, over the trochanter, to keep it pressed towards the neck of the bone; and thus they are held together until union is effected. I have, however, seen these fractures, when the girt is applied, do equally well in the extended position of the limb, as when placed over the double inclined plane, and in this respect I generally consult the patient's feelings.

" In fractures of the trochanter major a different plan must be pursued :—in this case, when the person is recumbent, the broken trochanter of the shaft will be found to have separated from the trochanter of the neck of the bone, and it is necessary to place a thick leather pad under the trochanter, to keep it elevated, so as to approximate the broken portions, or the bone will not unite. In Mr. Harris's relation of Mr. B———'s case, near Reading, not the least union was produced until this mode of treatment was adopted, although he had been kept in bed from July the 20th, to September the 11th.† I agree with our author that the fæces should be easily removed, and a portion of the mattress should draw out for that purpose; and this is readily done in any common bed."‡ 34.

It is surely unreasonable to charge Sir Astley Cooper with prejudice, because he will not, contrary to his experience and conviction, begin again attempts at bony union, which had repeatedly failed, and thus relinquish a mode of treatment which he found more beneficial than any other. But Sir Astley is unfairly accused of allowing no exceptions to his *general principle* of non-union by bone in the case in question. Whereas at page 142 of his treatise, he observes.

" ' I am never so wedded to any opinion as to be prevented from

---

* " This can be easily made, with two pieces of board, thus,  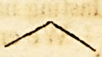 by any carpenter."

† " See case, page 159 of the Second Edition of my Work, and plate 30, for the bandages used."

‡ In another place Sir Astley makes the following observation :—

" When *forty-five* cases of united fracture of the neck of the os femoris are shewn against my *forty-four* of non-union, I will give up my general principle of non-union; and when five cases of fractures decidedly into the capsule are produced, of union by bone of this fracture (by surgical treatment,) without lameness or shortening of the limb, and the person walks better than he now does by the ligamentous union, I shall say that he who effected it deserves well of his country: but until that period arrives I shall continue of my present opinion.

" It is a reproach to some few of our English surgeons that the French, who formerly thought these cases admitted of ossific union, are now advertising a large reward for the best account of the cause that they do not unite by bone." 43.

trying, or wishing others to employ means which appear plausible or ingenious; and, therefore, this instrument ought to have a fair trial given to it."* 5.

Again at page 44, in order to enjoin caution, he has said—

" 'In every case, however, in which there is the smallest doubt whether it is a fracture within, or one external to the ligament, it would be proper to treat the case as if it were the fracture I shall hereafter describe, and which admits of ossific union.' " 5.

At page 43, we have the following remark.

" 'The cases in which *union might be produced are two :*—one in which the periosteum covering the neck of the thigh-bone is not torn through, which now and then happens; the other is when the head of the bone is broken, so that the cervix still remains in the acetabulum ;† but in neither of these cases, will the limb exhibit the shortened state which the fracture of the neck of the bone usually produces.' " 5.

Sir Astley Cooper has charged Mr. Earle with *censuring the practice of Guy's Hospital,* and thus making an indirect attack on the surgical school there. Mr. Earle denies the charge of "an attack on the *honour and credit* of Guy's Hospital"—a charge which we can see no where in Sir Astley's Appendix. This being a purely ethical question, however, we do not deem it necessary to go into it;—with *intentions* we having nothing to do. We only judge of actions, facts, and effects. But we do think that out of the present controversy one good may arise—a lesson to those who hope to fill conspicuous stations in medical society, not to begin by questioning the well-earned reputation of others, but by zealous and persevering endeavours to advance the science, and secure a lasting name for themselves.

We entered on the foregoing article with great reluctance, because nothing is so disagreeable to us as medical controversies. We have suffered ourselves to give no opinion unaccompanied by the facts or arguments on which it was founded, and to these last entirely we solicit the attention of our readers. A patient consideration of both sides of the question has led us to conclude that Mr. Earle has failed in every point of his criticism—and that all he has said on the possibility, probability, and propriety of bony union of the cervix femoris within the capsule, will not alter the general sentiments entertained on these points by surgeons before the appearance of "Practical Observations on Fractures."

---

* Mr. Hagerdorn's instrument for extending the thigh to produce bony union.

† "Mr. Brodie informed me of a case of that kind."

